# Occupational health promotion at a federal education institution: challenges and perspectives

**DOI:** 10.47626/1679-4435-2023-797

**Published:** 2023-04-18

**Authors:** Helinton Guedes de Mendonça, Thatiane Lopes Oliveira, Daniela Souza Santos De-Sá, Leila Conceição de Paula Miranda, Leonardo de Paula Miranda, Altamir Fernandes de Oliveira

**Affiliations:** 1 Programa de Pós-Graduação em Educação, Universidade Federal dos Vales do Jequitinhonha e Mucuri, Diamantina, MG, Brazil; 2 Enfermagem, Instituto Federal do Norte de Minas Gerais (IFNMG), Montes Claros, MG, Brazil; 3 Enfermagem, IFNMG, Januária, MG, Brazil; 4 Enfermagem, IFNMG, Almenara, MG, Brazil; 5 Programa de Pós-Graduação em Ciências da Saúde, Universidade Estadual de Montes Claros, Montes Claros, MG, Brazil

**Keywords:** federal government, health promotion, occupational health, governo federal, promoção da saúde, saúde do trabalhador

## Abstract

**Introduction:**

The Brazilian Federal Government developed the Occupational Health and Safety policy for the Federal Public Servant, supported by the following axes: health surveillance and promotion, health assistance for the civil servant, and medical surveillance expertise. As a federal public institution, the Federal Institute of Northern Minas Gerais (Instituto Federal do Norte de Minas Gerais) is responsible for implementing this policy.

**Objectives:**

This study aimed to identify the challenges and perspectives associated with the health care provided to the servants of the Federal Institute of Northern Minas Gerais.

**Methods:**

This was a documentary and field study, with a qualitative and quantitative approach, conducted using documentary research and semi-structured interviews. The collected data were submitted to descriptive and categorical content analyses.

**Results:**

The Federal Institute of Northern Minas Gerais still presents several problems in the consolidation and structuring of the Occupational Health and Safety policy for the Federal Public Servant. Among the main obstacles faced are the lack of governmental and institutional support and the precariousness of financial and human resources, mainly directed to the axis of health promotion and surveillance. The institution plans to conduct periodic medical examinations, the establishment of Internal Health Commissions for Public Servants, and the implementation of a mental health program.

**Conclusions:**

It is expected that the Federal Institute of Northern Minas Gerais will be able to perform better on the development of health policies and programs for its workers.

## Abstract (pt)

**Introdução:**

O Governo Federal criou a Política de Atenção à Saúde e Segurança do Trabalho do Servidor Público Federal, sustentada pelos seguintes eixos: vigilância e promoção à saúde, assistência à saúde do servidor e perícia em saúde. O Instituto Federal do Norte de Minas Gerais, enquanto instituição pública federal, detém o encargo de implementar a referida política.

**Objetivos:**

Este estudo objetivou identificar os desafios e perspectivas frente às ações de atenção à saúde do servidor no Instituto Federal do Norte de Minas Gerais.

**Métodos:**

Tratou-se de um estudo documental e de campo, de abordagem quali-quantitativa, realizado por meio de pesquisa documental e entrevista semiestruturada. Os dados coletados foram submetidos à análise descritiva e de conteúdo categorial.

**Resultados:**

O Instituto Federal do Norte de Minas Gerais ainda apresenta diversos problemas na consolidação e estruturação da Política de Atenção à Saúde e Segurança do Trabalho do Servidor Público Federal. Entre os principais obstáculos enfrentados, estão a falta de apoio governamental e institucional e a precariedade dos recursos financeiros e humanos, principalmente direcionados ao eixo de promoção e vigilância à saúde. A instituição planeja a realização de exames médicos periódicos, a criação de Comissões Internas de Saúde do Servidor Público e a implementação de um programa de saúde mental.

**Conclusões:**

Espera-se que o Instituto Federal do Norte de Minas Gerais possa melhor atuar no desenvolvimento de ações voltadas para a saúde dos servidores.

## INTRODUCTION

The relationship between health and work has always existed empirically across the human experience. In Brazil, labor regulation began in the 1940s with the Consolidation of Labor Laws, which focused on individual and collective relations.^1^ This legal framework led to the institutionalizing and orientation of workers’ health by countless normative documents, thus demonstrating that it is an area constantly evolving.^2-5^ Occupational health promotion has been studied and invested in by sectors that view it as a way to improve work performance.^6^

In order to consolidate actions in the area of occupational health and safety in the federal public service, the Occupational Health and Safety policy for the Federal Public Servant (Política de Atenção à Saúde e Segurança do Trabalho do Servidor Público Federal -PASS) was introduced in 2009, guided by actions of surveillance and health promotion, health care for the servant, and reports by medical experts. This policy is based on the interrelation between these axes, on the individual’s biopsychosocial approach, on multidisciplinary teamwork, on the evaluation of workplaces, and on epidemiological information.^7^

As a strategy for the implementation of the PASS proposals, the Integrated Subsystem of Servant Health Care (Subsistema Integrado de Atenção à Saúde do Servidor - SIASS) was also created, which is responsible for coordinating programs in the areas of health care, official investigation, and the promotion and monitoring of the health of public servants.^3^

Despite significant advances in the expansion of legal resources for its effectiveness, it was observed that the implementation of this policy faces many challenges. One of the most cited in the literature is the lack of planning, which shows an erroneous perception of occupational health, with actions focused on damages rather than on health promotion.^1,6^ Due to this lack of planning, no roles have been defined for the servants responsible for the effectiveness of the PASS, therefore servants and managers are not aware of it. Studies also show the need to expand the dialogue between health, labor, and management policies, and among the public servants themselves.^5,6,8^

The Federal Institute of Northern Minas Gerais (Instituto Federal do Norte de Minas Gerais - IFNMG), an institution of higher, basic and professional education, member of the Federal Public Administration (Administração Pública Federal - APF), is subject to the determinations of PASS. The health actions in the institution must be implemented in a systematic and programmatic way and executed by the health surveillance and promotion teams, in order to meet the PASS premises.

Thus, this study aimed to identify the challenges and perspectives facing the actions of health care for the workforce at IFNMG guided by PASS.

## METHODS

This was a documentary and field study, with a qualitative and quantitative approach, about the health care provided to staff at the IFNMG.

The IFNMG is an educational institution with multiple campuses and a decentralized organization. It is structured in several departments, directorates, and coordinators, to meet its objectives of teaching, research, and extension.

Data were collected between 05/05/2018 and 05/31/2018, and, for this purpose, documentary research and semi-structured interview were used.

The documentary research was used to gather information about policies and actions aimed at the health of public servants at IFNMG, by researching institutional regulatory documents, including the General Regulation, the Internal Regulations, and the document on the Institutional Program of Health Care and Quality of Life of Public Servants (PIASQV),^9-12^ among others. The semi-structured interview was prepared by the authors themselves and covered questions according to the proposed objective.

Six employees participated in the study, intentionally selected to occupy strategic positions regarding the institution’s healthcare actions. They were four members of the institutional health care body (coordinator, physician, psychologist, and occupational safety technician), the director of human resources management, representing the body in which the health care functions are allocated, and a professional from the financial sector, to explain the resources invested in workers’ health.

The interviews were conducted individually, by a single researcher, after adherence to the Free and Informed Consent Form. These meetings took place in the institution itself and lasted 30 minutes on average.

The interviews were recorded and transcribed, and their data were submitted to categorical content analysis, using analytical description and subsequent interpretation.

The study was approved by a duly recognized Research Ethics Committee (under no. 2,636,457) and by the institution involved, and it was developed according to the guidelines determined by Resolution no. 466/2012 of the Brazilian National Health Council.^13^

## RESULTS AND DISCUSSION

The IFNMG has a decentralized organization, with a rectory and 11 campuses distributed in the mesoregions of the North of the state of Minas, in the Jequitinhonha Valley, in the Mucuri Valley, and also in part of the Northwest of Minas. The realities of these campuses are diverse, with similar characteristics and demands in some aspects, but different in terms of regional specificities. While one of them has a history of more than 60 years of experience in offering technical courses and a more consolidated structure, others were established only a few years ago, some even without their own headquarters, offering only high school technical education. This regional coverage is the result of a process of institutional expansion aimed at meeting the education and training needs of the local and regional society.^14^

In investigating the effectiveness of PASS in this institution, four thematic categories were constructed:

- institutional bodies in the health care of public servants;
- health programs and actions aligned to the PASS;
- challenges in the implementation of health actions according to PASS; and
- institutional proposals in face of the reality experienced.

### INSTITUTIONAL BODIES IN THE HEALTH CARE OF PUBLIC SERVANTS

The PASS establishes that the attributions related to the development of health programs and actions are up to the personnel management body of these bodies. The Directorate of People Management (A Diretoria de Gestão de Pessoas - DGP) of IFNMG, located in the rectory, recognizes and has assumed its role in the health care of servers, as attested in the following speech: “The issue of health promotion [...] represents one of the strategic aspects in the area of People Management [...], and, therefore, we [...] have invested in the composition of a team under the conditions we have” (E1).

However, this body also acknowledges the difficulties when fulfilling its responsibilities: “There are a number of limitations, including personnel shortages, resource constraints, and a lack of political clarity” (E1).

To put into practice what is established in the PASS, the DGP has a Health Assistance and Quality of Life Coordination (Coordenação de Assistência à Saúde e Qualidade de Vida do Servidor (CASQV) and a Workplace Safety Center (Núcleo de Segurança do Trabalho -NST).^11^

The CASQV is composed of an administrative assistant, who articulates the actions of this coordination, a psychologist, who also participates in these actions and performs individual consultations, and a physician, who performs the medical examinations of the servants and takes part in some health promotion activities. The NST, on the other hand, is comprised of an occupational safety engineer, who performs occupational safety examinations and deals with occupational additional requests, and an occupational safety technician, who manages occupational health and safety.^11^

Since they are located exclusively in the rectory, the activities developed by these bodies are mainly directed at the rectory staff. On the campuses, the Coordinators of People Management (Coordenadorias de Gestão de Pessoas - CGP) are responsible for supporting and subsidizing the actions proposed by CASQV.^10^ Such reality is confirmed by the following report:

We cannot effectively reach all campuses, visit them, meet people, talk to them, and make the Coordination’s action reach them in a massive way, we work here thinking about the policies, applying them a little to the rectory and trying to overflow a little to the campuses (E1).

Some campuses have health professionals who, despite their functions being focused on student assistance, are also partners of CASQV, collaborating in activities of this Coordination, when requested:

We also have a very close connection with the health professionals on the campuses, including doctors, psychologists, social workers, dentists, nutritionists, all these professionals… We have a relationship, and we are often working together to develop actions on campuses (E2).

The SIASS has the Integrated System of Human Resources Administration - Health (Sistema Integrado de Administração de Recursos Humanos/Saúde (SIAPE-Health) as a platform for its activities, a system designed to automate the health information of the federal public servant.^15^ Feeding this platform is essential for the successful implementation of the PASS, especially with regard to health promotion and prevention actions.^16^

It is important to point out that the SIASS unit at IFNMG does not have a health surveillance and promotion team as recommended by the PASS, therefore it uses the same professionals that comprise the CASQV and the NST as a workforce.^11^

### HEALTH PROGRAMS AND ACTIONS ALIGNED TO THE AXES OF THE PASS

As previously reported, the PASS is guided by the axes of health expertise, health care, and health surveillance and promotion. It was verified that, regarding the health expertise actions, IFNMG fulfills its role by providing medical care to its employees, at the SIASS unit in the rectory and also at the other campuses where there is a medical professional.

According to Jackson Filho & Ponce,^8^ for a long time the health actions in the institutions were restricted to conducting medical reports. For the authors, performing only these actions means leaving aside other peculiarities that involve the work and health issues present in the institutions. This emphasis on actions such as medical-legal reports to the detriment of health promotion actions can contribute to the risk of SIASS becoming only a medical-legal control model.^5^

Concerning healthcare actions, we verified that they are not conducted directly by health professionals within the IFNMG. The Brazilian Federal Administration offers supplementary health benefits to its servants, intended for the medical and dental care of servants and dependents, being a benefit shared between the APF and the servants. Furthermore, the Brazilian public Unified Health System (SUS), offered to all Brazilian citizens, is also considered a healthcare resource in the context of the PASS.^7^

The inability to directly provide health care is a reality at several federal institutions, as this would require a proper structure in terms of material and human resources, which most institutions lack. The policy only establishes the benefit of supplementary health without specifying the institutions’ responsibility in terms of assistance.

In the axis of health promotion and surveillance actions, we verified a predominance of campaigns, events, and lectures related to health and safety at work. These actions are conducted in specific periods of the year in a non-systematic way, which can be seen in the excerpts of the following statements: “There is no systematic, programmatic nature of the performance, but some actions have already become part of our annual calendar, such as vaccination campaign and, informative campaigns on certain topics” (E1).

Health promotion actions have improved over the years.^17^ It is always important to point out that these actions should focus on strengthening people’s health, encouraging healthy habits and consequently preventing lifestyle-related diseases.^18,19^ Therefore, they have great importance within any institution.

One of the possible obstacles to the implementation of this axis at IFNMG is that the regulatory instruments of PASS do not determine which actions should be developed or establish a requirement for its implementation^5^. Health promotion and prevention actions usually do not produce immediately visible results, which leads to the question of whether PASS has really fulfilled its role in improving the health of employees.^16^

### CHALLENGES IN THE IMPLEMENTATION OF HEALTH ACTIONS ACCORDING TO PASS

PASS represents a promising policy, but the effectiveness of its practices involves many challenges. Based on the report below, we observed that one of these challenges is the lack of financial resources: “A recurring remark from the team is that without financial resources they cannot do anything, right? So with few resources they do something, but if there are not enough, it discourages the team to present projects and solutions, with the perspective that the projects will not be sponsored” (E1).

The APF bodies are responsible for providing the necessary resources to implement health surveillance and promotion programs.^20^ However, in the IFNMG budget there are no specific resources assigned for these actions. Since its implementation, CASQV and NST have never had any specific resources, detached from the institution’s budget, to conduct their health activities, as verified in the following statement: “We understand that without financial resources we cannot offer the necessary services and actions to our workers. We have already asked the Board of Directors for these resources two, three times, but in the current scenario, they haven’t been granted yet. So today CASQV has no resources of its own” (E2).

The lack of financial resources to conduct health actions is a common challenge among agencies and institutions within the federal public service. The lack of clarity in the definition of budget allocations for health care for employees has generated difficulties in implementing these actions.^5,21,22^

The lack of governmental and institutional support is also a challenge. The dissonance between the creation of policies and the availability of resources for their implementation has already been evaluated as a lack of governmental support.^16^ As for the institution, we notice the need for greater prioritization of investments in health promotion, as also described by other authors.^1,5,6^

Complementary to the lack of governmental support is the deficiency of normative instruments, which do not oblige federal public institutions to develop actions to promote occupational health and safety. What exists is a recommendation. Public institutions follow the norms established for private companies, but they are not obligatory. About this obstacle, it is interesting to analyze: “I see that the public sector has a great inconsistency, it often creates the laws and impose a reality for the private sector, while it is the largest body and has the largest number of employees, it leaves much to be desired in this aspect in its own administration” (E6).

The absence of mandatory preventive actions can have several negative consequences, among them: decentralized actions without guidelines; lack of infrastructure and specialized technical personnel; low level of satisfaction among employees; among others.^23^

Just as there is a need for more effective action by the government and the institution, there is also a need for greater protagonism of the institutional actors. The employees must understand that they need to be protagonists of health actions, participating in institutional actions related to the health and safety of the employee. In this sense, an interviewee reports: “The servant must understand that he needs it, he needs to worry about his health as soon as possible. [...] Then, we need to provoke the managers in the sense that they see that this need exists” (E6).

Gonçalves et al.^24^ consider the employee as the main actor in the work process related to health care initiatives related to PASS. They also argue that the construction of this look at health requires the integrative action of the tripod institution, individuals, and collectivity.

Another challenge mentioned concerns the need for training of professionals who deal with health actions within the SIASS unit of IFNMG: “The SIASS is structured to perform various activities. [...] And these activities often have a very high degree of complexity, and there has not been [...] for some time now, a training of the SIASS professional staff, this is a criticism at the Brazilian level.” (E3).

The formation and training of professionals to deal with the health programs and actions that integrate the PASS are foreseen in the policy itself.^25^

The lack of personal resources is also a major hindering factor in IFNMG. This lack of human resources is not limited to the health department, located in the rectory, but also extends to all campuses, as verified in the speeches:

In the work safety area there is what we call SESMT, which is the Specialized Service in Safety Engineering and Occupational Medicine. This system is formed by a multi-professional team. [...] So, these professionals from the safety area form a multi-professional team. [...] We are very far from forming this multidisciplinary team (E4).

We have already discussed the need for an occupational physician and psychiatrist for ICD medical leaves. We also need a social worker, because today withdrawals are only handled at the level of medical expertise, and the server does not have a psychologist or a social worker, to monitor medical leaves (E2).

The difficulty in hiring new professionals through public exams was verified, due to a government restriction on the number of public servants. It is known that it is the responsibility of both the Union and the local agencies and institutions to ensure the provision of human resources necessary for the effectiveness of the programs.^7,20,25^

Another major challenge faced by the institution is the multi-campuses format, which makes it difficult for servers from other campuses to access the actions developed by CASQV and NST, as can be seen in the speech:

We are an institution of more than 1,300 servants with 11 units, so we are still figuring out the best format for the CASQV to operate, to try and verify if this centralized structure in the rectory is feasible to meet all our public needs, even with the professionals I mentioned, or if these services, or part of them, need to be suddenly decentralized to the campuses so that this monitoring can be done on-site (E2).

Gonçalves et al.^24^ also reported the need for a greater “capillarity” of the actions developed at the university concerning its geographic spaces. One option for the institution would be web-based programs. Studies have shown that these programs bring significant results in promoting worker health due to the advantages of mobile access to information. In addition, there are benefits for the employees, who can participate at their convenience, as well as savings for the institution when compared to face-to-face programs.^26^

### INSTITUTIONAL PROPOSALS IN VIEW OF THE REALITY EXPERIENCED

The IFNMG bodies responsible for staff health have been working to make the institution’s health programs and actions a reality. Among some proposals, there are periodic medical examinations, the creation of the Internal Commissions for Public Servant Health (ICPSH), and the implementation of mental health programs.

Several normative and institutional documents stipulate the need for periodic medical exams.^10,11,27^ However, although specific resources have already been made available by the Federal Government to implement the exams, no companies have expressed interest in the bidding process. Therefore, this resource is no longer included in the institution’s budget.

The proposed exams are a strategy to promote the health of public servants, through periodic monitoring of the health status of individuals and the recording of this information in the SIAPE-Health database for follow-up, monitoring, and construction of the epidemiological profile of public servants. Given its importance, these exams must be conducted. An alternative for IFNMG, which does not have health services with its own laboratory and infrastructure, would be to perform these exams through private health plans since the government offers the benefit of supplementary health care.

Another important initiative for the promotion of public health in the context of the PASS is the creation of the CISSP, which aims to propose actions aimed at promoting health and humanization at work. With shared management, the CISSP aims to expand the autonomy of public employees so that they can contribute to regulating their activities, and negotiating changes in the environment and work organization within the administration.^7,25^ The implementation of the CISSP is already foreseen in regulatory documents that deal with the health of public servants.^11,12^ This can directly influence the workers’ health since the workers’ health problems are linked to several types of hazards in the work environment. The CISSP will allow an integrated approach to this environment, overcoming a fragmented approach to occupational health and acting on the risk factors to which workers are exposed.^28^

The establishment of a program of mental health actions is also established in the PASS, regulated by Administrative Rule 1.261, May 5, 2010. According to this standard, the mental health procedures adopted must be in line with the public policies on mental health and workers’ health.^29^

The mental health care provided in the institution consists of psychological care, with no care or therapeutic purpose, and non-systematic health actions. We verified that the IFNMG needs to develop more consistent and effective actions, according to government policies.^29^ As in other educational institutions, mental disorders are among the main causes of sick leave in the agencies. This reality awakens the need for greater attention to mental health issues at work. In order to minimize absences and illness, it is imperative to identify the magnitude of the problem and its potential causes.^30^

The lack of funding brings uncertainties about the progress of the actions that are currently conducted at IFNMG. While there is a consensus about the need for evolution of institutional programs and actions, the lack of resources prevents such initiatives from advancing.

A limiting factor of this study was the unilateral investigation. The view of the servers as users assisted by the institution’s health bodies was not evaluated. The results of this analysis allow a greater extrapolation of this investigation, with the evaluation of the health of the servant from the servant’s perspective. It is suggested that this issue be addressed in further studies, which will possibly bring significant contributions to the planning of institutional health programs.

## CONCLUSIONS

The attention to the health and safety of staff at IFNMG is hindered by a lack of human and financial resources. We emphasize the need for increased governmental and institutional support, especially directed to the axis of health promotion and surveillance established by PASS.

Despite the difficulties and challenges encountered, the institution is expected to be more proactive in developing actions that improve the health of its employees, with the formulation of appropriate policies and prioritizing resources within the institutional framework.

## References

[1] Martins MIC, Oliveira SS, Andrade ET, Strauzz MC, Castro LCF, Azambuja A (2017). Brazil’s policy on healthcare for government workers: players,
paths and challenges. Cien Saude Colet.

[2] Brasil (2022). Supremo Tribunal Federal (STF). Constituição da República Federativa do
Brasil;.

[3] Brasil, Presidência da República, Casa Civil (2009). Decreto nº 6.833, de 29 de abril de 2009. Institui o Subsistema
Integrado de Atenção à Saúde do Servidor - SIASS
e o Comitê Gestor de Atenção à Saúde do
Servidor.

[4] Brasil, Presidência da República, Casa Civil (2011). Decreto nº 7.602, de 7 de novembro de 2011. Dispõe sobre a
Política Nacional de Segurança e Saúde no Trabalho -
PNSST.

[5] Zanin FDC, Künzle LA, Perna PDO, Muntsch SMA (2015). Política de atenção à saúde e
segurança do trabalho do servidor público no
Brasil. Univ Soc.

[6] De Souza ZB, Dos Reis LM (2013). Entre o atender e o ser atendido: políticas em
saúde para o trabalhador do serviço
público. CPST.

[7] Brasil, Ministério do Planejamento, Secretaria de Recursos Humanos (2010). Subsistema Integrado de Atenção à Saúde do
Servidor (SIASS).

[8] Jackson JM, Ponce TB (2017). O papel dos agentes de recursos humanos na
implementação da Política de Atenção
à Saúde e Segurança do Trabalho do Servidor
Público Federal (PASS). Rev Serv Público.

[9] Instituto Federal do Norte de Minas Gerais (IFNMG) (2011). Regimento Geral.

[10] Instituto Federal do Norte de Minas Gerais (IFNMG) (2013). Regimento Interno dos Campi.

[11] Instituto Federal do Norte de Minas Gerais (IFNMG) (2013). Programa Institucional de Assistência à Saúde e
Qualidade de Vida do Servidor.

[12] Instituto Federal do Norte de Minas Gerais (IFNMG) (2013). Plano de Desenvolvimento Institucional (PDI) 2014 - 2018.

[13] Brasil, Conselho Nacional de Saúde (2012). Resolução nº 466, de 12 de dezembro de 2012.

[14] Da Silva PF (2015). A expansão da educação superior e o trabalho
docente no Instituto Federal do Norte de Minas Gerais. 2015.

[15] Brasil, Ministério do Planejamento, Subsistema Integrado de Atenção à Saúde
do Servidor (SIASS) (2013). Manual Operacional do SIAPE Saúde. Módulo: Perícia
Oficial em Saúde. Perfil Gestor do Sistema na Unidade SIASS.

[16] Ferreira AS (2014). Análise do processo de implementação das
ações de promoção e prevenção em
saúde preconizadas na Política de Atenção
à Saúde do Servidor (PASS): o caso da Unidade SIASS/Unb.
2014.

[17] Povlsen L, Borup I (2015). Health Promotion: A developing focus area over the
years. Scand J Public Health.

[18] Hanson A (2004). Hälsopromotion i arbetslivet.

[19] Mæland JG (1999). Forebyggende helsearbejde i teori og praksis.

[20] Brasil, Ministério do Planejamento (2013). Portaria Normativa nº 3 de 25 de março de 2013a. Institui as
diretrizes gerais de promoção da saúde do servidor
público federal, que visam orientar os órgãos e
entidades do Sistema de Pessoal Civil da Administração Federal
- SIPEC.

[21] Santos JAC (2016). Saúde do trabalhador no serviço público federal:
desafios para uma Política de Atenção à
Saúde e Segurança do Trabalho no contexto de um hospital
universitário.

[22] Mesquita TRP (2015). Trabalho e saúde: um desafio para a gestão de pessoas da
Universidade Federal do Tocantins.

[23] De Araújo AP (2014). Política de Atenção à Saúde do
Servidor Público Federal: um estudo sobre a implantação
do Subsistema de Atenção à Saúde do Servidor -
SIASS (2009-2013).

[24] Gonçalves SD, De Medeiros RB, Taissuke ASN, De Melo PB, De Carvalho APO, De Menezes Rolim FA (2016). Promoção e vigilância à saúde
dos servidores públicos: a experiência da Universidade Federal
do Ceará. Rev Psicol.

[25] Brasil (2010). Portaria Normativa nº 03 de 07 de maio de 2010. Estabelece
orientações básicas sobre a Norma Operacional de
Saúde do Servidor - NOSS.

[26] Dos Santos NC, Santos LS, Camelier FWR, Maciel RRBT, Portella DDA (2017). Technologies applied to occupational health promotion: a
systematic review. Rev Bras Med Trab.

[27] Brasil, Presidência da República, Casa Civil (2009). Decreto nº 6.856 de 25 de maio de 2009. Regulamenta o art. 206-A da Lei
no 8.112, de 11 de dezembro de 1990 - Regime Jurídico Único,
dispondo sobre os exames médicos periódicos de
servidores.

[28] Ferreira AP, Grams MT, Erthal R, Girianelli VR, Oliveira MHB (2018). Literature review on working environment hazards relative to the
working conditions and impact on workers’ health. Rev Bras Med Trab.

[29] Brasil (2010). Portaria Normativa nº 1.261 de 5 de maio de 2010. Institui os
Princípios, Diretrizes e Ações em Saúde Mental
que visam orientar os órgãos e entidades do Sistema de Pessoal
Civil - SIPEC da Administração Pública Federal sobre a
saúde mental dos servidores.

[30] Bastos MLA, Da Silva Junior GB, Domingos ETC, Araújo RMOD, Santos ALD (2018). Afastamentos do trabalho por transtornos mentais: um estudo de
caso com servidores públicos em uma instituição de
ensino no Ceará, Brasil. Rev Bras Med Trab.

